# The Relationship Between Chronotype and Depression Among Adolescents: The Mediating Role of Bedtime Procrastination and the Moderating Role of Parental Psychological Control

**DOI:** 10.3390/bs16081327

**Published:** 2026-08-03

**Authors:** Wenqi Zhang, Ziqing Wang, Qian Wang, Yingying Zhu

**Affiliations:** 1 Faculty of Psychology, Tianjin Normal University, Tianjin 300387, China; 13655876802@163.com (W.Z.); psy_ziqingwang@126.com (Z.W.); qianwang_psy@126.com (Q.W.); 2 Key Research Base of Humanities and Social Sciences of the Ministry of Education, Academy of Psychology and Behavior, Tianjin Normal University, Tianjin 300387, China; 3 Tianjin Social Science Laboratory of Students’ Mental Development and Learning, Tianjin 300387, China

**Keywords:** chronotype, depression, bedtime procrastination, parental psychological control, adolescents

## Abstract

Depression has been a pressing mental health concern among contemporary adolescents. Accumulating evidence suggests that individuals’ circadian preference—chronotype—may be associated with adolescent depression, yet the potential mediating and moderating roles within this association have received limited attention in existing research. The present study aimed to examine the relationship between chronotype and depression among adolescents, as well as the mediating role of bedtime procrastination and the moderating role of parental psychological control. A cross-sectional study was conducted among 4324 adolescents (63.9% female; mean age = 16.24 ± 0.92 years) using a questionnaire survey method. All participants completed self-report measurements, including the Composite Morningness Questionnaire (CMQ), Bedtime Procrastination Scale (BPS), Patient Health Questionnaire-9 (PHQ-9), and Parental Psychological Control Scale (PPCS). The results showed that chronotype was significantly and negatively associated with depression among adolescents (*β* = −0.461, *p* < 0.001). Bedtime procrastination partially mediated the association between chronotype and adolescent depression (*β* = −0.257, 95%CI [−0.283, −0.232]), with a mediating effect size of 55.75%. Moreover, parental psychological control moderated the direct association between chronotype and depression (*β* = −0.043, 95%CI [−0.070, −0.016]), as well as the association between bedtime procrastination and depression (*β* = 0.120, 95%CI [0.092, 0.148]). Specifically, high levels of parental control were linked to a more pronounced relationship between chronotype and depression, as well as a stronger association between bedtime procrastination and depression. These findings advance our understanding of how chronotype is related to adolescent depression, and suggest that multilevel interventions targeting sleep hygiene practices and family dynamics may be promising for reducing depressive risk among vulnerable adolescents. However, given the cross-sectional design, future longitudinal or experimental studies are needed to establish temporal precedence and further test these associations.

## 1. Introduction

Depression is one of the most prevalent mental health problems among adolescents (Singh et al., 2023). During this developmental period, both its prevalence and symptom severity increase significantly, driven by rapid physiological and psychological maturation as well as substantial changes in social and academic environments (Hankin et al., 2015). Epidemiological evidence indicates that the global prevalence of depression among adolescents ranges from 5% to 24% (Askari et al., 2024; Shorey et al., 2022), with estimates in China ranging from 10% to 24.6% (Yue et al., 2015; S. J. Zhou et al., 2020). Depressive symptoms exert widespread and persistent adverse effects on adolescents’ academic performance, social functioning, and self-concept development. Moreover, they substantially increase the risk of chronic mental health disorders (e.g., major depressive disorder, anxiety disorders) if unresolved into adulthood (Thapar et al., 2022). In severe cases, adolescent depression may even lead to suicidal ideation and non-suicidal self-injury behaviors (Zubrick et al., 2017). Given the high prevalence and detrimental consequences of adolescent depression, identifying relevant risk factors and understanding the processes involved are critical for informing evidence-based prevention and targeted intervention strategies to promote adolescent mental health.

### 1.1. Chronotype and Depression in Adolescents

Among the numerous risk correlates of adolescent depression, chronotype has emerged as a robust and clinically meaningful construct, attracting increasing research attention in recent years (Bauducco et al., 2020; Haraden et al., 2017; Horne et al., 2019; Shi et al., 2019). Chronotype refers to an individual’s circadian preference, reflecting inter-individual differences in the timing of sleep–wake patterns and daily activity peaks (Taylor & Hasler, 2018). It is commonly classified into morning, intermediate, and evening types (Rique et al., 2014). Morning-type individuals typically wake early and reach peak alertness and performance in the first half of the day (Taillard et al., 2004). In contrast, evening-type individuals exhibit delayed sleep–wake schedules, with optimal cognitive and physical functioning occurring in the late evening (Adan et al., 2012; Montaruli et al., 2017). A growing body of research evidence indicates that chronotype is associated with depression (Zou et al., 2025) and can negatively predict depression in adolescence (F. Wang et al., 2025). According to the two-process model of sleep regulation (Dijk & Lockley, 2002), the human’s sleep–wake cycle is governed by the interaction between homeostatic sleep pressure and circadian processes. Circadian dysregulation—namely, misalignment between an individual’s internal circadian rhythm and external social schedules—can readily lead to sleep disruptions and complaints, which in turn constitute important proximal antecedents of emotional disorders such as depression and anxiety (Bautista et al., 2025; Ye et al., 2025). Cross-sectional studies have consistently shown that individuals with an evening preference exhibit a higher prevalence of emotional problems compared to morning or intermediate types (Zhang et al., 2023). A recent study by Mao et al. (2024) further found that evening chronotype was robustly associated with depression in adolescents. From the perspective of social jetlag, eveningness is associated with disrupted emotional regulation and a higher likelihood of depressive and anxious symptoms, which may reflect the conflict between an individual’s internal biological clock and social or work schedules (Lee et al., 2011; Wittmann et al., 2006). Despite accumulating evidence linking chronotype to adolescent depression, the mediating and moderating roles within this association remain understudied. To address this gap, the present study explored a moderated mediation model with a large sample of Chinese adolescents, examining whether bedtime procrastination mediates this association and whether parental psychological control moderates it.

### 1.2. The Potential Mediating Role of Bedtime Procrastination

Bedtime procrastination is defined as going to bed later than intended without any external constraints (Kroese et al., 2014). It differs from typical procrastination, which involves a delay in aversive tasks, in that sleep is generally not regarded as aversive (Kroese et al., 2016). Previous research has indicated that there is a close relationship between chronotype and bedtime procrastination in both adolescents and college students (Carciofo & Cheung, 2025; Z. Y. Liu et al., 2025). For example, compared to morning-type adolescents, those with evening-type chronotypes report significantly higher levels of bedtime procrastination (Kadzikowska-Wrzosek, 2020). This pattern has been further corroborated by follow-up studies, which indicate that evening types engage in more frequent bedtime procrastination, and this behavior becomes even more pronounced on non-school days or when self-regulatory resources are relatively abundant (Pu et al., 2022). Additionally, researchers have found that evening-type individuals typically exhibit weaker pre-sleep self-control and greater difficulty resisting immediately rewarding activities (e.g., using mobile phones or watching videos), which in turn increases their likelihood of bedtime procrastination (Exelmans & van den Bulck, 2017; Kamphorst et al., 2018). Beyond its association with chronotype, bedtime procrastination is also closely related to depressive symptoms. A recent meta-analysis has confirmed a significant positive association between bedtime procrastination and depression among adolescents (Azeem et al., 2026). Consistent with this, both cross-sectional and longitudinal studies demonstrate that adolescents and young adults who engage in more frequent bedtime procrastination report higher levels of depressive symptoms (Cui et al., 2021; Guo et al., 2020). As a maladaptive pre-sleep behavior, bedtime procrastination contributes to insufficient sleep duration and poor sleep quality (Zhu et al., 2022), both of which are well-established proximal risk factors for depression (Soltani et al., 2023). Furthermore, sleep insufficiency has been linked to disrupted functional connectivity between the striatum and the default mode and somatosensory networks (L. Wang et al., 2018), and such sleep-related neural dysregulation may be associated with vulnerability to depression. Additionally, according to the temporal self-regulation theory (Cameron, 2010), evening-type adolescents tend to prioritize immediate gratification over delayed sleep benefits. This self-regulatory conflict may not only foster bedtime procrastination but also amplify their vulnerability to depressive symptoms. Therefore, based on the evidence outlined above, we propose that bedtime procrastination would mediate the relationship between chronotype and depression in adolescents.

### 1.3. The Potential Moderating Role of Parental Psychological Control

Parenting style is a central contextual factor in adolescent development (Darling & Steinberg, 2017). Among various parenting dimensions, parental psychological control—characterized by intrusive strategies such as guilt induction and love withdrawal—has been consistently associated with maladaptive psychological outcomes (Barber et al., 1994; Shek & Law, 2014). Previous research has shown that adolescents who perceive higher levels of parental control are more likely to develop an evening chronotype (Eberhardt & Gunn, 2019) and that parental psychological control is associated with both chronotype and depression (Dong et al., 2022). Neuroimaging studies have also revealed that high psychological control disrupts prefrontal–limbic connectivity during emotional processing, thereby increasing individuals’ depression risk (Marusak et al., 2018). Grounded in the two-process model of sleep regulation, eveningness is associated with circadian misalignment and emotional dysregulation (Dijk & Lockley, 2002). High parental psychological control may further restrict autonomy and undermine emotional adjustment, thereby amplifying the link between evening chronotype and depressive symptoms (Hare et al., 2015; Marusak et al., 2018; Pérez et al., 2021), whereas lower levels may provide a protective context that buffers the negative emotional consequences associated with an evening chronotype. Accordingly, based on relevant theory and empirical studies, we assumed that parental psychological control would play a moderating role in the association between chronotype and depression in adolescents.

Existing research has also shown that parental psychological control is associated with adolescents’ bedtime procrastination behavior (Eberhardt & Gunn, 2019; Deng et al., 2024). It has been found that evening-type individuals tend to exhibit weaker pre-sleep self-control and greater difficulty resisting immediately rewarding activities, which may be associated with a greater tendency toward bedtime procrastination (Exelmans & van den Bulck, 2017; Kamphorst et al., 2018). According to self-determination theory (Deci & Ryan, 2012), excessive parental psychological control may hinder the development of adolescents’ autonomy and self-regulatory capacities (Yılmaz & Büyükcebeci, 2019). Given that self-regulation deficits are a risk factor for bedtime procrastination (Kühnel et al., 2018; Kadzikowska-Wrzosek, 2020), adolescents exposed to higher levels of psychological control may be particularly vulnerable to bedtime procrastination. Empirical evidence also shows a positive association between parental psychological control and bedtime procrastination, mediated by negative affect and reduced self-control (Deng et al., 2024). Conversely, lower levels of psychological control may preserve adolescents’ regulatory resources and emotional resilience, mitigating the negative impact of bedtime procrastination on mental health. Building on these findings, we proposed that parental psychological control would be a moderator in the relationship between chronotype and depression within the mediating role of bedtime procrastination. In other words, the negative association between chronotype and bedtime procrastination would be stronger among adolescents with high parental psychological control and weaker among those with low parental psychological control; similarly, the positive association between bedtime procrastination and depression would be stronger among adolescents with high parental psychological control and weaker among those with low parental psychological control.

### 1.4. The Present Study

The present study focused on adolescents as the research subjects and aimed to investigate the relationship between chronotype and adolescent depression, with a particular focus on the mediating and moderating roles in this association. Specifically, the first objective was to examine the mediating role of bedtime procrastination in the link between chronotype and depression. The second objective was to assess the moderating role of parental psychological control within this mediation model. Based on previous theoretical and empirical evidence, we proposed the following hypotheses: (1) Chronotype would be negatively associated with adolescents’ depression; (2) bedtime procrastination would mediate the association between chronotype and depression; and (3) parental psychological control would moderate both the direct association between chronotype and depression and the indirect association via bedtime procrastination.

## 2. Methods

### 2.1. Participants and Procedure

A questionnaire-based cross-sectional study was conducted from September to November 2024 at a public high school in Hefei, Anhui Province, China. The school was selected based on accessibility and its willingness to participate and comprised a total of 58 classes across Grades 10 to 12. Using a cluster sampling approach, we recruited all students from randomly selected classes across these grades, with intact classes as the sampling units. A total of 4324 students were invited to participate. Among the participants, 63.9% were female, and 36.1% were male, with an average age of 16.24 ± 0.92 years. The vast majority (80.9%) were rural residents, and the remaining 19.1% were urban residents. 94.4% of the adolescents came from intact families and 5.6% from single-parent families. Approximately 94.0% of the adolescents reported that they were not the only child. Most adolescents (84.9%) reported an average family economic status, while 9.5% and 5.6% reported very good and very poor economic conditions, respectively. In addition, 16.8% of the adolescents’ fathers and 33.5% of their mothers had a primary school education or below. Prior to data collection, all participants and their primary caregivers provided informed consent. Under the supervision of well-trained school teachers, all participants completed self-report questionnaires that assessed sociodemographic information, chronotype, bedtime procrastination, depression, and parental psychological control. During this session, they were informed that participation was voluntary and that they could withdraw from the study at any time. The study was reviewed and approved by the Ethics Committee of Tianjin Normal University and was conducted in accordance with the Declaration of Helsinki.

### 2.2. Measurements

#### 2.2.1. Chronotype

Chronotype was measured using the Composite Morningness Questionnaire (CMQ; Lu et al., 2016). This questionnaire consists of 13 items (e.g., “Think about the pace of life that makes you feel best”; “If you could plan your entire day, when would you get up?”). These items were clearly divided into three factors: morning activity, morning influence, and night situation. Items 1, 2, and 7 use a 5-point scoring system, while the remaining items employ a 4-point scale. The overall score spans from 13 to 55. Higher CMQ scores indicate a stronger morning preference, and lower CMQ scores indicate a stronger evening preference. The Chinese version of the CMQ has demonstrated satisfactory construct validity and internal consistency in samples of Chinese adolescents (Shi et al., 2019). In this study, the Cronbach’s α coefficient for the scale was 0.81.

#### 2.2.2. Depression

Participants’ depression was assessed by the 9-item Patient Health Questionnaire (PHQ-9), developed by Kroenke et al. (2001), which is widely used for rapid screening in both clinical and community settings. It has been validated to have good reliability and consistency among Chinese adolescents (Hu et al., 2014). The scale is a dimension scale with 9 items (e.g., “Little interest or pleasure in doing things”; “Poor appetite or overeating”). Responses were made on a four-point scale, ranging from 0 (“completely absent”) to 3 (“almost every day”). The total score ranges from 0 to 27, with higher scores indicating more severe depression. In this study, Cronbach’s α coefficient for the scale was 0.92.

#### 2.2.3. Bedtime Procrastination

Bedtime procrastination was measured by the Chinese version of the Bedtime Procrastination Scale (BPS), revised by Ma et al. (2021). It is a single-dimension scale with 9 items (e.g., “My bedtime is later than I plan”; “When it’s time to go to bed, I’m often engaged in other things”). Responses were made on a five-point Likert scale, ranging from 1 (“never”) to 5 (“always”). Items 2, 3, 7, and 9 were inversely scored. The BPS score was calculated as the average of all item ratings after reverse-coding the negatively worded items. Higher scores indicate higher levels of bedtime procrastination. Previous studies have demonstrated the satisfactory validity and reliability of the scale among Chinese adolescents (Mao et al., 2022). In this study, Cronbach’s α coefficient for the scale was 0.81.

#### 2.2.4. Parental Psychological Control

The Parental Psychological Control Scale (PPCS) developed by Q. Wang et al. (2007) was used to measure adolescents’ perceived level of parental psychological control. This scale consists of 18 self-report items, which can be divided into three dimensions: guilt induction (e.g., “My parents tell me that I should feel guilty when I do not meet their expectations”), love withdrawal (e.g., “My parents act cold and unfriendly if I do something they do not like”), and authority assertion (e.g., “My parents tell me that what they want me to do is the best for me and I should not question it”). All items were rated on a five-point Likert scale ranging from 1 (“completely inconsistent”) to 5 (“completely consistent”). The PPCS score was calculated as the average of all items, ranging from 1 to 5, with higher scores indicating greater levels of parental psychological control. This scale has demonstrated satisfactory construct validity and internal reliability in samples of Chinese adolescents (Xu et al., 2020). In this study, the Cronbach’s α coefficient for the scale was 0.95.

#### 2.2.5. Covariates

Based on prior research linking sociodemographic factors to adolescent depression and sleep-related outcomes (H. S. Liu & Li, 2025; T. Tang et al., 2024; Zhong, 2023), we included gender, age, residence, family type, only-child and family economic status, and parental educational levels as covariates to control for potential confounding effects.

### 2.3. Statistical Analysis

All data analyses were conducted using SPSS 25.0 (IBM) and the PROCESS macro (version 3.5; Hayes, 2013). First, Harman’s single-factor test and the Unmeasured Latent Method Construct approach (ULMC) were performed to assess potential common method bias, thereby providing a preliminary check on the validity of the self-reported data (Podsakoff et al., 2003; D. Tang & Wen, 2020). Descriptive statistics and Pearson correlation analyses were then conducted to examine the means, standard deviations, and bivariate relationships among the study variables. All continuous variables were standardized prior to analyses to reduce multicollinearity. To test the hypothesized mediation model, PROCESS Macro Model 4 was employed to examine the mediating role of bedtime procrastination in the relationship between chronotype and depression. Furthermore, PROCESS Macro Model 59 was used to test the moderated mediation model, in which parental psychological control was specified as a moderator of all three paths in the mediation model. The significance of the indirect effects was evaluated using a bias-corrected bootstrap approach with 5000 resamples. From this, the standard error (*SE*) and 95% confidence interval (95%CI) of the parameter estimate were obtained. An indirect effect is considered statistically significant if the 95%CI does not include zero. Beyond p-values and 95% confidence intervals for statistical inference, standardized regression coefficients and model *R*^2^ values are also reported throughout the results to evaluate the practical magnitude of the observed associations.

## 3. Results

### 3.1. Test of Common Method Bias (CMB)

Given that all variables were self-reported by adolescents, we employed Harman’s single-factor test and the Unmeasured Latent Method Construct (ULMC) approach to assess common method variance (Podsakoff et al., 2003; D. Tang & Wen, 2020). The results of Harman’s single-factor test revealed that eight factors had eigenvalues greater than 1, with the first factor accounting for only 28.99% of the total variance (less than the 40% threshold; H. Zhou & Long, 2004). Furthermore, the ULMC analysis showed that adding a common method latent factor resulted in minimal changes in model fit (ΔCFI = 0.016, ΔTLI = 0.008, ΔRMSEA = −0.001), all well below the critical thresholds (increases in CFI and TLI greater than 0.10 and decreases in RMSEA greater than 0.05; Wen et al., 2018). Taken together, these results indicate that common method bias is not a serious concern in the present study.

### 3.2. Descriptive Statistics and Correlation Analysis

The means, standard deviations, and Pearson correlations of the main variables are presented in Table 1. The results showed that chronotype was significantly and negatively correlated with depression (*r* = −0.470, *p* < 0.01) and bedtime procrastination (*r* = −0.645, *p* < 0.01). Bedtime procrastination was significantly and positively correlated with depression (*r* = 0.538, *p* < 0.01) as well. In addition, parental psychological control was significantly and positively correlated with higher depression (*r* = 0.442, *p* < 0.01) and greater bedtime procrastination (*r* = 0.333, *p* < 0.01), respectively.

### 3.3. Testing for the Mediation Model

**Figure 1:**
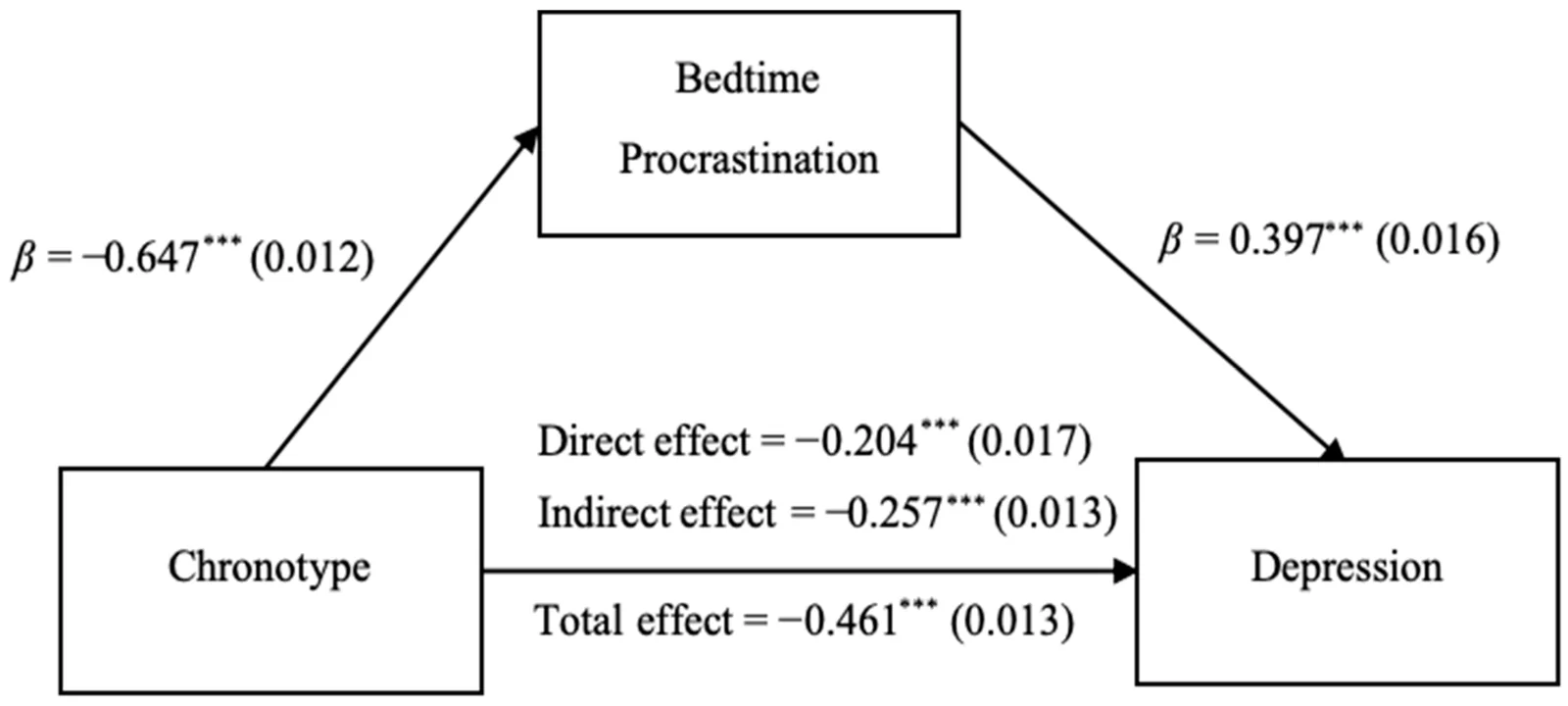
The mediating effect of bedtime procrastination on the relationship between chronotype and depression. Standard errors are presented in parentheses. *** *p* < 0.001.

Hayes’s PROCESS (Model 4) was utilized to test the mediation model, with chronotype as the independent variable, bedtime procrastination as the mediator, and depression as the dependent variable; sociodemographic variables, including gender, age, residence, only-child status, family type, family economic status, and parental educational levels, were included as covariates. The mediation analysis results are shown in Table 2 and Figure 1. Chronotype exhibited a significant negative association with both bedtime procrastination (*β* = −0.647, *p* < 0.001) and depression (*β* = −0.204, *p* < 0.001). Bedtime procrastination showed a significant positive association with depression (*β* = 0.397, *p* < 0.001). The direct effect of chronotype on depression was significant (*β* = −0.204, *p* < 0.001). The indirect effect via bedtime procrastination (*β* = −0.257, 95%CI [−0.283, −0.232]) was also significant, accounting for 55.75% of the total effect. These results indicate that bedtime procrastination partially mediated the association between chronotype and depression among adolescents.

### 3.4. Testing for the Moderation Effect of Parental Psychological Control

The macro PROCESS (Model 59) was adopted to test the moderated mediation model, with chronotype as the independent variable, bedtime procrastination as the mediator, and depression as the dependent variable; parental psychological control was specified as the moderator (as shown in Table 3). The results showed that chronotype was negatively associated with both bedtime procrastination (*β* = −0.602, *t* = −50.261, 95%CI [−0.625, −0.578]) and depression (*β* = −0.168, *t* = −10.885, 95%CI [−0.198, −0.134]). Parental psychological control was positively associated with both bedtime procrastination (*β* = 0.161, *t* = 13.448, 95%CI [0.138, 0.185]) and depression (*β* = 0.249, *t* = 19.558, 95%CI [0.224, 0.274]). The interaction between chronotype and parental psychological control was significantly associated with depression (*β* = −0.043, *t* = −3.151, 95%CI [−0.070, −0.016]), indicating that parental psychological control moderated the direct association between chronotype and depression, whereas the chronotype × parental psychological control interaction was not significantly associated with bedtime procrastination (*β* = 0.011, *t* = 1.077, 95%CI [−0.009, 0.031]). The interaction between bedtime procrastination and parental psychological control was also significant (*β* = 0.120, *t* = 8.497, 95%CI [0.092, 0.148]), suggesting that parental psychological control moderated the indirect association via bedtime procrastination. The conditional indirect effects are presented in Table 4, with an index of moderated mediation of −0.078, 95%CI [−0.103, −0.053], indicating that the magnitude of the negative indirect effect increased as levels of parental psychological control rose.

**Figure 2:**
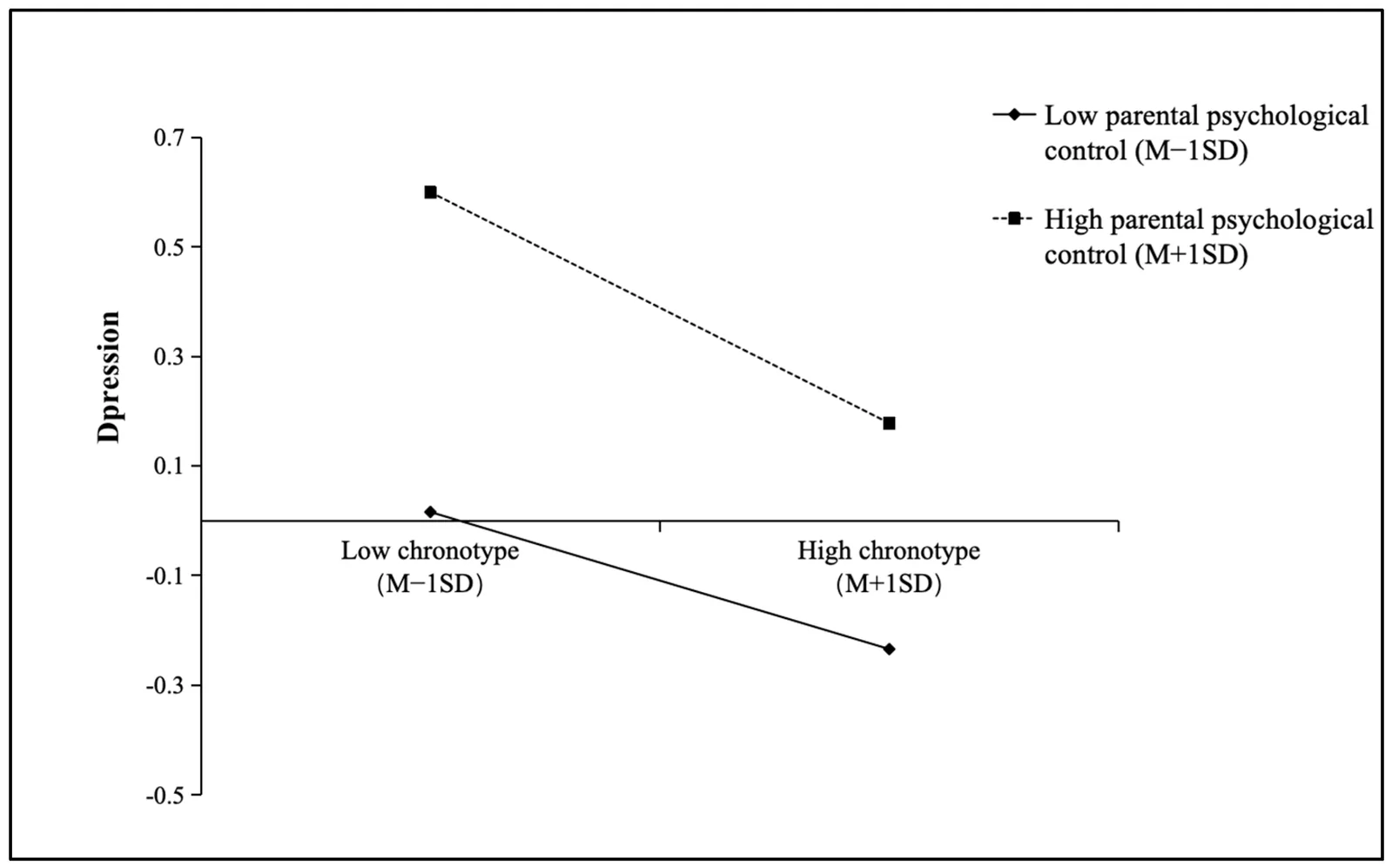
The moderating effect of parental psychological control on the association between chronotype and adolescent depression.

**Figure 3:**
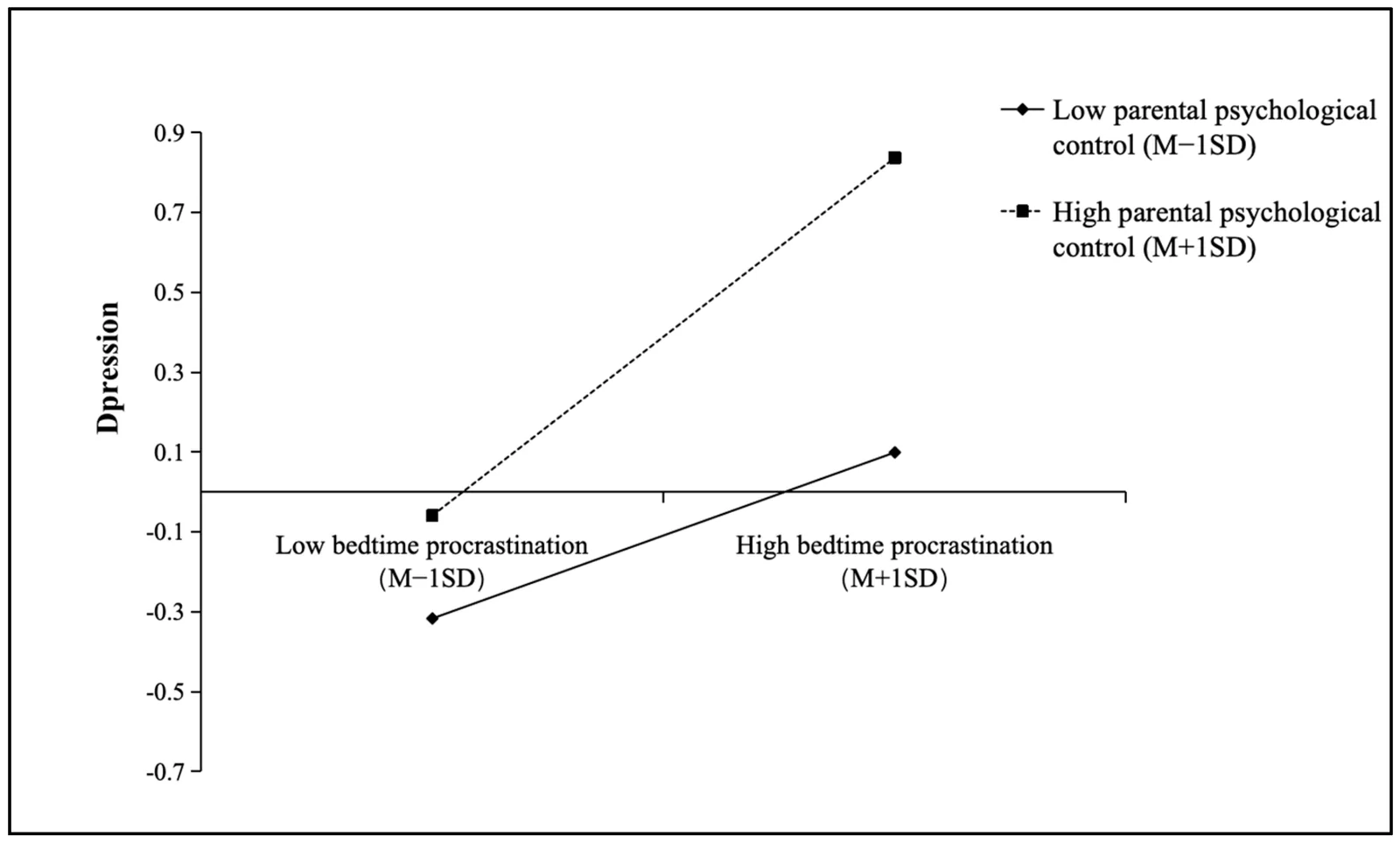
The moderating effect of parental psychological control on the association between bedtime procrastination and adolescent depression.

To further elucidate the moderated mediation effect, simple slope analyses were conducted to decompose the interaction effect at low (−1 SD) and high (+1 SD) levels of parental psychological control. As illustrated in Figure 2, chronotype was negatively associated with depression, with a stronger association under high parental psychological control (*β_simple_* = −0.211, *t* = −10.535, *p* < 0.001) than under low parental psychological control (*β_simple_* = −0.125, *t* = −5.903, *p* < 0.001). Similarly, bedtime procrastination was positively associated with depression, with a stronger association in the high parental psychological control group (*β_simple_* = 0.448, *t* = 21.357, *p* < 0.001) compared to the low parental psychological control group (*β_simple_* = 0.208, *t* = 9.867, *p* < 0.001; see Figure 3). These results indicate that as the level of parental psychological control increases, the association between bedtime procrastination and depression gradually strengthens. Supplementary Johnson–Neyman significance tests and percentile-based conditional indirect effect analyses further confirmed that these findings were consistent across the full range of parental psychological control (see Supplementary Figures S1 and S2 and Table S1).

## 4. Discussion

In the present study, we employed a large sample to investigate the relationship between chronotype and depression among adolescents, as well as the potential mediating and moderating roles within this association. The results extend previous findings on the connection between chronotype and emotional problems in adolescents and also clarify how and when chronotype is related to adolescents’ depression. Moreover, the results of this study may provide comprehensive guidance for educators and mental health professionals in developing targeted, family-centered interventions aimed at reducing bedtime procrastination and mitigating the risk of depression among adolescents.

We found that chronotype was significantly negatively associated with adolescent depression, confirming Hypothesis 1. The finding aligns with a substantial body of cross-sectional and longitudinal studies demonstrating that evening-type individuals are more likely to report elevated depression, with this risk increasing over time (Haraden et al., 2017; Van den Berg et al., 2018). One plausible explanation is that eveningness is associated with delayed circadian timing, which may lead to poorer sleep quality and impaired emotion regulation and in turn corresponds to a reduced capacity to cope with depressive experiences. Furthermore, evening-oriented adolescents face persistent misalignment between their internal circadian rhythm and early school schedules; this chronic social jetlag may increase fatigue and perceived stress while undermining social functioning and self-efficacy, ultimately linking eveningness to greater vulnerability to depression (Lee et al., 2011). Notably, the present results should be interpreted within the specific educational context of the sample. The study was conducted in a public senior high school in Anhui province, southern China, where students are subject to academic demands associated with preparation for the National College Entrance Examination. Given this context-specific sample, the generalizability of these findings to other populations such as students in less academically demanding settings or those from different regional and cultural backgrounds requires further investigation. Taken together, these findings provide empirical support for the two-process model of sleep regulation (Dijk & Lockley, 2002) and suggest that circadian-aligned strategies such as promoting morning light exposure, encouraging regular sleep–wake schedules, and, where feasible, delaying school start times may help reduce the risk of depression among adolescents.

Moreover, our study extends prior literature by demonstrating that bedtime procrastination played a mediating role in the relationship between chronotype and depression in adolescents, which supports Hypothesis 2. This finding is consistent with previous research showing that high levels of bedtime procrastination are associated with depressive symptoms in adolescence (Pu et al., 2022). Research has characterized bedtime procrastination as a form of self-regulation failure, involving impaired behavioral control and difficulty resisting short-term rewards (Lian et al., 2018). Consistent with this perspective, the present study found that individuals with a stronger preference for eveningness tend to exhibit weaker pre-sleep behavioral control and a greater tendency to engage in immediately rewarding activities (e.g., using electronic devices) during late evening hours, thereby increasing the likelihood of delayed bedtimes (Exelmans & van den Bulck, 2017). Bedtime procrastination was also closely linked to insufficient sleep duration and poor sleep quality (Zhu et al., 2022; Kelly & El-Sheikh, 2014), both of which are established risk factors for depression. Therefore, bedtime procrastination may serve as a key mediator in the relationship between chronotype and depression, particularly among individuals with evening chronotypes. Interventions aimed at reducing bedtime procrastination (e.g., priority-setting training, self-regulation skills, and fostering future time perspective) could be integrated into daily routines to help adolescents mitigate the negative impact of evening preference on depressive symptoms.

The present study also revealed that parental psychological control moderated the direct association between chronotype and depression and the association between bedtime procrastination and depression, but not the association between chronotype and bedtime procrastination, partially supporting Hypothesis 3. These moderating effects are in line with prior evidence identifying parental psychological control as a contextual correlate of adolescent depressive symptoms (Chyung et al., 2022). Drawing on ecological systems theory (Bronfenbrenner, 1979) and self-determination theory (Deci & Ryan, 2012), the family, as a proximal microsystem, may amplify or buffer individual risk factors. Specifically, high levels of parental psychological control may restrict adolescents’ autonomy and intensify external evaluative standards (Soenens & Vansteenkiste, 2010; Costa et al., 2016), thereby exacerbating the emotional dysregulation that evening-type adolescents experience due to social jetlag (Haraden et al., 2017). For adolescents who already engage in bedtime procrastination, high parental psychological control may also strengthen the association between bedtime procrastination and depressive symptoms, whereas low levels may provide a supportive family context that buffers the emotional vulnerability associated with both eveningness preference and bedtime procrastination. The non-significant moderation of the chronotype–bedtime procrastination link suggests that this association is more tied to stable circadian trait characteristics and thus less susceptible to family contextual influences. Collectively, these findings suggest that adolescent depressive symptoms may be associated with the interplay between family environment and individual circadian characteristics as well as sleep-related behaviors. High parental psychological control may act as an environmental stressor that may amplify these associations, whereas low levels may serve as a protective factor.

In addition, these findings should be interpreted within the sociocultural context of Chinese collectivist societies. In such cultural settings, parents tend to adopt a highly involved and controlling role in child development, and this parenting style is often perceived as normative or even necessary and may be internalized by adolescents to some extent (Chao, 1994; Soenens et al., 2018). Consequently, parental psychological control may not only function as an external stressor but also become internalized by adolescents, acquiring stronger self-referential meaning and amplifying its emotional consequences (Soenens & Vansteenkiste, 2010; M. Wang et al., 2014). This cultural characteristic provides additional support for the present finding that parental psychological control primarily intensified the emotion-related pathways to depressive symptoms, while exerting relatively limited influence on the biologically grounded association between chronotype and bedtime procrastination. Alongside these cultural insights, the current findings have important implications for social and educational practice. For instance, school-based mental health programs could integrate psychoeducational components aimed at promoting autonomy-supportive parenting and sleep health literacy among both parents and adolescents. In parallel, community-based preventive strategies might prioritize improving parent–adolescent communication, regulating pre-sleep screen use, and fostering healthy sleep hygiene practices. Integrating these multi-level interventions may be beneficial for reducing adolescents’ vulnerability to depression and promoting their long-term psychological well-being.

## 5. Limitations and Future Directions

Several limitations of this study should be acknowledged when interpreting the findings. First, the cross-sectional design of this study prevents us from establishing the temporal ordering of constructs in the proposed mediation model, as all variables were assessed at a single time point, and reverse or reciprocal associations (e.g., elevated depressive symptoms may be associated with greater bedtime procrastination) cannot be ruled out. Future longitudinal studies are needed to clarify the temporal ordering of these variables, and experimental designs are required to draw causal inferences. Second, the measures in the present study were collected via self-report questionnaires, which may introduce recall and social desirability biases to some extent. Future studies could incorporate more objective and sensitive indicators to improve data reliability. Third, we did not assess objective or subjective sleep parameters (e.g., sleep duration and quality), chronic stressful life events, or participants’ mental health history. Future research could incorporate such assessments to further investigate the relationships between chronotype, bedtime procrastination, and depressive symptoms. Fourth, though parental psychological control significantly moderated the direct association between chronotype and depression, its relatively small effect size warrants caution in interpreting its practical significance. Finally, the study sample was primarily drawn from a public senior high school in Anhui Province, southern China, which may limit the generalizability of the findings to adolescents from other regions or educational systems with different sociocultural contexts. In addition, as participants were recruited through intact school classes, the dataset has an inherent class-level nested structure. However, class-level identifiers were not retained in the anonymized dataset to protect participant confidentiality, which precluded the use of multilevel modeling or cluster-robust standard errors. Although the present study focused on associations among individual-level psychological variables, future research could recruit more diverse samples from multiple schools across broader geographic regions and educational settings and, where appropriate, employ multilevel analytical approaches to account for the nested data structure, thereby enhancing the representativeness and robustness of the findings.

## 6. Conclusions

This study examined the relationship between chronotype and depression among adolescents, with a particular focus on the mediating role of bedtime procrastination and the moderating role of parental psychological control. Overall, the results indicated that bedtime procrastination partially mediated the association between chronotype and depression, such that adolescents with eveningness preferences were more likely to show higher levels of bedtime procrastination, which in turn was associated with increased depressive symptoms. Furthermore, parental psychological control was found to significantly moderate the direct link between chronotype and depression and the link between bedtime procrastination and depression, but not the chronotype–bedtime procrastination link. That is, the associations of chronotype and bedtime procrastination with adolescents’ depression were both stronger under high levels of parental psychological control. From a practical perspective, these findings suggest that combining sleep intervention programs (e.g., chronotype-tailored sleep strategies, reducing bedtime procrastination) and family-based parenting guidance (e.g., mitigating maladaptive parenting practices) may be promising for preventing and alleviating depression among adolescents.

## Figures and Tables

**Table 1:** Means, standard deviation, and correlations between main variables (*N* = 4324).

Variables	1	2	3	4	5	6	7	8	9	10	11	12
1. Gender	–											
2. Age	0.012	–										
3. Residence	−0.004	−0.110 **	–									
4. Only child	0.093 **	−0.005	0.024	–								
5. Family type	−0.005	0.043 **	0.002	−0.220 **	–							
6. Family economic status	−0.040 **	−0.022	0.054 **	−0.028	0.159 **	–						
7. Father’s educational level	−0.026	−0.043 **	0.097 **	−0.012	0.044 **	0.088 **	–					
8. Mother’s educational level	−0.003	−0.097 **	0.157 **	0.026	−0.032 *	0.082 **	0.332 **	–				
9. Chronotype	0.092 **	−0.076 **	−0.052 **	−0.001	0.034 *	0.041 **	0.035 *	0.030	–			
10. Depression	−0.074 **	0.044 **	0.004	−0.006	−0.052 **	−0.107 **	−0.064 **	−0.064 **	−0.470 **	–		
11. Bedtime procrastination	−0.018	0.048 **	−0.008	0.005	−0.058 **	−0.066 **	−0.068 **	−0.056 **	−0.645 **	0.538 **	–	
12. PPC	0.082 **	0.022	0.025	0.022	−0.027	−0.076 **	−0.023	−0.024	−0.282 **	0.442 **	0.333 **	–
Mean (*M*)									38.910	3.310	2.597	1.953
Standard deviations (*SD*)									6.053	4.382	0.686	0.809

Notes: PPC, parental psychological control. All categorical variables presented in the table were dummy-coded with 0 as the reference group: gender (0 = female, 1 = male); residence (0 = rural, 1 = urban); only child (0 = no, 1 = yes); family type (0 = single-parent family, 1 = two-parent family); family economic status (0 = poor, 1 = average and above); father’s educational level (0 = primary school and below, 1 = junior high school and above); mother’s educational level (same coding as father’s); * *p* < 0.05, ** *p* < 0.01.

**Table 2:** The mediation analysis of bedtime procrastination in the relationship between chronotype and depression among adolescents.

Predictors	Equation 1: Bedtime Procrastination				Equation 2: Depression			
	*β*	*SE*	*t*	95% CI	*β*	*SE*	*t*	95% CI
Gender	0.084	0.024	3.473 **	[0.037, 0.132]	−0.105	0.026	−3.999 ***	[−0.157, −0.054]
Age	−0.009	0.013	−0.685	[−0.034,0.016]	0.007	0.014	0.542	[−0.019, 0.034]
Residence	−0.087	0.030	−2.907 **	[−0.146, −0.028]	0.017	0.033	0.528	[−0.047, 0.081]
Only child	−0.025	0.050	−0.498	[−0.124, 0.074]	−0.036	0.054	−0.665	[−0.143, 0.071]
Family type	−0.100	0.047	−2.593 **	[−0.175, −0.024]	−0.043	0.042	−1.023	[−0.124, 0.039]
Family economic status	−0.115	0.038	−2.241 *	[−0.216, −0.014]	−0.303	0.056	−5.429 ***	[−0.412, −0.193]
Father’s educational level	−0.085	0.051	−2.566 **	[−0.149, −0.020]	−0.043	0.036	−1.213	[−0.113, 0.027]
Mother’s educational level	−0.043	0.026	−1.624	[−0.094, 0.009]	−0.053	0.029	−1.868	[−0.109, 0.003]
Chronotype	−0.647	0.012	−55.336 ***	[−0.670, −0.624]	−0.204	0.017	−12.298 ***	[−0.236, −0.171]
Bedtime procrastination					0.397	0.016	24.113 ***	[0.365, 0.430]
*R* ^2^	0.651				0.569			
*F*	351.919 ***				207.027 ***			

Notes: Categorical covariates were coded using the same scheme as specified in Table 1. *β*: Standardized coefficients. *R*^2^ denotes the proportion of variance explained by each regression equation. * *p* < 0.05, ** *p* < 0.01, *** *p* < 0.001.

**Table 3:** The moderated mediation effects of chronotype on adolescents’ depression.

Predictors	Equation 1: Bedtime Procrastination				Equation 2: Depression			
	*β*	*SE*	*t*	95% CI	*β*	*SE*	*t*	95% CI
Gender	0.048	0.024	1.989 *	[0.001, 0.095]	−0.138	0.025	−5.609 ***	[−0.186, −0.090]
Age	−0.009	0.012	−0.713	[−0.033, 0.016]	0.012	0.013	0.971	[−0.013, 0.037]
Residence	−0.093	0.029	−3.160 **	[−0.151, −0.035]	−0.014	0.030	−0.458	[−0.073, 0.045]
Only child	−0.029	0.049	−0.598	[−0.126, 0.067]	−0.083	0.050	−1.650	[−0.182, 0.016]
Family type	−0.096	0.038	−2.554 *	[−0.170, −0.022]	−0.052	0.039	−1.343	[−0.127, 0.024]
Family economic status	−0.057	0.05	−1.492	[−0.174, 0.024]	−0.234	0.052	−4.542 ***	[−0.335, −0.133]
Father’s educational level	−0.083	0.032	−2.567 **	[−0.146, −0.020]	−0.049	0.033	−1.487	[−0.114, 0.016]
Mother’s educational level	−0.039	0.026	−1.512	[−0.090, 0.012]	−0.041	0.026	−1.545	[−0.092, 0.011]
Chronotype	−0.602	0.012	−50.261 ***	[−0.625, −0.578]	−0.168	0.015	−10.885 ***	[−0.198, −0.134]
PPC	0.161	0.012	13.448 ***	[0.138, 0.185]	0.249	0.013	19.558 ***	[0.224, 0.274]
Chronotype × PPC	0.011	0.010	1.077	[−0.009, 0.031]	−0.043	0.014	−3.151 **	[−0.070, −0.016]
Bedtime Procrastination					0.328	0.016	21.098 ***	[0.298, 0.358]
Bedtime Procrastination × PPC					0.120	0.014	8.497 ***	[0.092, 0.148]
*R* ^2^	0.668				0.651			
*F*	316.357 ***				243.621 ***			

Notes: PPC, parental psychological control. Categorical covariates were coded using the same scheme as described in Table 1. *β*: Standardized coefficients. *R*^2^ denotes the proportion of variance explained by each regression equation. * *p* < 0.05, ** *p* < 0.01, *** *p* < 0.001.

**Table 4:** Conditional indirect effects of the moderated mediation model at different levels of parental psychological control.

Parental Psychological Control	Effect	*SE*	95%CI	
			Boot LLCI	Boot ULCI
*M* − 1 *SD*	−0.134	0.013	−0.160	−0.109
*M*	−0.212	0.012	−0.235	−0.190
*M* + 1 *SD*	−0.290	0.021	−0.331	−0.250

Notes: Boot LLCI and Boot ULCI refer to the lower and upper limits of the 95% confidence interval of the indirect effects estimated by the percentile bootstrap method with deviation correction, respectively.

## Data Availability

Data are available from the corresponding author upon reasonable request.

## Supplementary Materials

The following supporting information can be downloaded at: https://www.mdpi.com/article/10.3390/bs16081327/s1, Figure S1: Johnson–Neyman plot of the conditional direct association between chronotype and depression across levels of parental psychological control; Figure S2: Johnson–Neyman plot of the conditional association between bedtime procrastination and depression across levels of parental psychological control; Table S1: Conditional indirect effects at different percentiles of parental psychological control.

## Abbreviations

The following abbreviations are used in this manuscript:

PPC	Parental psychological control
PPCS	Parental psychological control scale
BPS	Bedtime procrastination scale
CMQ	Composite Morningness Questionnaire
PHQ-9	9-Item Patient Health Questionnaire
M	Mean
SD	Standard deviation
CI	Confidence interval
SE	Standard error

## References

[B1-behavsci-16-01327] Adan A., Archer S. N., Hidalgo M. P., Di Milia L., Natale V., Randler C. (2012). Circadian typology: A comprehensive review. Chronobiology International.

[B2-behavsci-16-01327] Askari M. S., Olfson M., Belsky D. W., Breslau J., Keyes K. M. (2024). The influence of the great recession on adolescent major depressive episodes and treatment in the United States: An interrupted time series analysis. Journal of Adolescent Health.

[B3-behavsci-16-01327] Azeem O., Sulaiman F., Haidong Z. (2026). Bedtime procrastination and psychological distress in university students: A systematic review and meta-analysis of their association. Frontiers in Psychology.

[B4-behavsci-16-01327] Barber B. K., Olsen J. E., Shagle S. C. (1994). Associations between parental psychological and behavioral control and youth internalized and externalized behaviors. Child Development.

[B5-behavsci-16-01327] Bauducco S., Richardson C., Gradisar M. (2020). Chronotype, circadian rhythms and mood. Current Opinion in Psychology.

[B6-behavsci-16-01327] Bautista J., Hidalgo-Tinoco C., Di Capua Delgado M., Viteri-Recalde J., Guerra-Guerrero A., López-Cortés A. (2025). The gut–brain–circadian axis in anxiety and depression: A critical review. Frontiers in Psychiatry.

[B7-behavsci-16-01327] Bronfenbrenner U. (1979). The ecology of human development: Experiments by nature and design.

[B8-behavsci-16-01327] Cameron L. D. (2010). Temporal self-regulation theory: Towards a more comprehensive understanding of health behaviour. Health Psychology Review.

[B9-behavsci-16-01327] Carciofo R., Cheung R. Y. (2025). Eveningness and procrastination: An exploration of relationships with mind wandering, sleep quality, self-control, and depression. European Journal of Investigation in Health, Psychology and Education.

[B10-behavsci-16-01327] Chao R. K. (1994). Beyond parental control and authoritarian parenting style: Understanding Chinese parenting through the cultural notion of training. Child Development.

[B11-behavsci-16-01327] Chyung Y. J., Lee Y. A., Ahn S. J., Bang H. S. (2022). Associations of perceived parental psychological control with depression, anxiety in children and adolescents: A meta-analysis. Marriage & Family Review.

[B12-behavsci-16-01327] Costa S., Cuzzocrea F., Gugliandolo M. C., Larcan R. (2016). Associations between parental psychological control and autonomy support, and psychological outcomes in adolescents: The mediating role of need satisfaction and need frustration. Child Indicators Research.

[B13-behavsci-16-01327] Cui G., Yin Y., Li S., Chen L., Liu X., Tang K., Li Y. (2021). Longitudinal relationships among problematic mobile phone use, bedtime procrastination, sleep quality and depressive symptoms in Chinese college students: A cross-lagged panel analysis. BMC Psychiatry.

[B14-behavsci-16-01327] Darling N., Steinberg L. (2017). Parenting style as context: An integrative model. Interpersonal development.

[B15-behavsci-16-01327] Deci E. L., Ryan R. M. (2012). Self-determination theory. Handbook of Theories of Social Psychology.

[B16-behavsci-16-01327] Deng Y., Ye B., Yang Q. (2024). “Why don’t you go to bed on time”? Parental psychological control and bedtime procrastination among Chinese adolescents. Children and Youth Services Review.

[B17-behavsci-16-01327] Dijk D. J., Lockley S. W. (2002). Invited Review: Integration of human sleep-wake regulation and circadian rhythmicity. Journal of Applied Physiology.

[B18-behavsci-16-01327] Dong X., Liang G., Li D., Liu M., Yin Z., Li Y., Zhang T., Pan C. W. (2022). Association between parental control and depressive symptoms among college freshmen in China: The chain mediating role of chronotype and sleep quality. Journal of Affective Disorders.

[B19-behavsci-16-01327] Eberhardt K., Gunn H. E. (2019). 0775 Family matters: Parental monitoring, chronotype, and daily rhythmicity. Sleep.

[B20-behavsci-16-01327] Exelmans L., van den Bulck J. (2017). Bedtime, shuteye time and electronic media: Sleep displacement is a two-step process. Journal of Sleep Research.

[B21-behavsci-16-01327] Guo J., Meng D., Ma X., Zhu L., Yang L., Mu L. (2020). The impact of bedtime procrastination on depression symptoms in Chinese medical students. Sleep and Breathing.

[B22-behavsci-16-01327] Hankin B. L., Young J. F., Abela J. R., Smolen A., Jenness J. L., Gulley L. D., Technow J. R., Gottlieb A. B., Cohen J. R., Oppenheimer C. W. (2015). Depression from childhood into late adolescence: Influence of gender, development, genetic susceptibility, and peer stress. Journal of Abnormal Psychology.

[B23-behavsci-16-01327] Haraden D. A., Mullin B. C., Hankin B. L. (2017). The relationship between depression and chronotype: A longitudinal assessment during childhood and adolescence. Depression and Anxiety.

[B24-behavsci-16-01327] Hare A. L., Szwedo D. E., Schad M. M., Allen J. P. (2015). Undermining adolescent autonomy with parents and peers: The enduring implications of psychologically controlling parenting. Journal of Research on Adolescence.

[B25-behavsci-16-01327] Hayes A. F. (2013). Introduction to mediation, moderation, and conditional process analysis.

[B26-behavsci-16-01327] Horne C. M., Watts A. L., Norbury R. (2019). The influence of subjective sleep quality on the association between eveningness and depressive symptoms. Biological Rhythm Research.

[B27-behavsci-16-01327] Hu X., Zhang Y., Liang W., Zhang H., Yang S. (2014). Reliability and validity of the patient health quetionnaire-9 in Chinese adolescents. Sichuan Mental Health.

[B28-behavsci-16-01327] Kadzikowska-Wrzosek R. (2020). Insufficient sleep among adolescents: The role of bedtime procrastination, chronotype and autonomous vs. controlled motivational regulations. Current Psychology.

[B29-behavsci-16-01327] Kamphorst B. A., Nauts S., De Ridder D. T. D., Anderson J. H. (2018). Too depleted to turn in: The relevance of end-of-the-day resource depletion for reducing bedtime procrastination. Frontiers in Psychology.

[B30-behavsci-16-01327] Kelly R. J., El-Sheikh M. (2014). Reciprocal relations between children’s sleep and their adjustment over time. Developmental Psychology.

[B31-behavsci-16-01327] Kroenke K., Spitzer R. L., Williams J. B. (2001). The PHQ-9: Validity of a brief depression severity measure. Journal of General Internal Medicine.

[B32-behavsci-16-01327] Kroese F. M., De Ridder D. T., Evers C., Adriaanse M. A. (2014). Bedtime procrastination: Introducing a new area of procrastination. Frontiers in Psychology.

[B33-behavsci-16-01327] Kroese F. M., Evers C., Adriaanse M. A., de Ridder D. T. D. (2016). Bedtime procrastination: A self-regulation perspective on sleep insufficiency in the general population. Journal of Health Psychology.

[B34-behavsci-16-01327] Kühnel J., Syrek C. J., Dreher A. (2018). Why don’t you go to bed on time? A daily diary study on the relationships between chronotype, self-control resources and the phenomenon of bedtime procrastination. Frontiers in Psychology.

[B35-behavsci-16-01327] Lee H. J., Rex K. M., Nievergelt C. M., Kelsoe J. R., Kripke D. F. (2011). Delayed sleep phase syndrome is related to seasonal affective disorder. Journal of Affective Disorders.

[B36-behavsci-16-01327] Lian Y., Li S., Liu J., Sun X. (2018). Self-regulation and bedtime procrastination: The role of self-regulation skills and chronotype. Personality and Individual Differences.

[B37-behavsci-16-01327] Liu H. S., Li Z. W. (2025). Differences in the effects of intergenerational family support types on adolescent depression in urban and rural areas: Evidence from the 2020 CFPS data. Social Work and Management.

[B38-behavsci-16-01327] Liu Z. Y., Chen Y. J., Bian T. Y., Xiang Y., Yuan R. M., Yang H. X. (2025). Associations among smartphone addiction, chronotype, and bedtime procrastination: Bayesian network analysis and pilot intervention. Current Psychology.

[B39-behavsci-16-01327] Lu L., Wang X., Tang X. (2016). Scales related to sleep and sleep disorders.

[B40-behavsci-16-01327] Ma X., Zhu L., Guo J., Zhao Y., Fu Y., Mu L. (2021). Reliability and validity of the Chinese version of the bedtime procrastination behavior scale in college students. Journal of Chinese Clinical Psychology.

[B41-behavsci-16-01327] Mao B., Chen S., Wei M., Luo Y., Liu Y. (2022). Future time perspective and bedtime procrastination: The mediating role of dual-mode self-control and problematic smartphone use. International Journal of Environmental Research and Public Health.

[B42-behavsci-16-01327] Mao B., Xie Z., Liu M., Gong Y., Wang H., Yang S., Liao M., Xiao T., Tang S., Wang Y., Yang Y.-D. (2024). Associations of chronotype with anxiety, depression and insomnia among general adult population: A cross-sectional study in Hubei, China. Journal of Affective Disorders.

[B43-behavsci-16-01327] Marusak H. A., Thomason M. E., Sala-Hamrick K., Crespo L., Rabinak C. A. (2018). What’s parenting got to do with it: Emotional autonomy and brain and behavioral responses to emotional conflict in children and adolescents. Developmental Science.

[B44-behavsci-16-01327] Montaruli A., Galasso L., Caumo A., Cè E., Pesenti C., Roveda E., Esposito F. (2017). The circadian typology: The role of physical activity and melatonin. Sport Sciences for Health.

[B45-behavsci-16-01327] Pérez J. C., Huerta P., Rubio B., Fernández O. (2021). Parental psychological control: Maternal, adolescent, and contextual predictors. Frontiers in Psychology.

[B46-behavsci-16-01327] Podsakoff P. M., MacKenzie S. B., Lee J. Y., Podsakoff N. P. (2003). Common method biases in behavioral research: Acritical review of the literature and recommended remedies. Journal of Applied Psychology.

[B47-behavsci-16-01327] Pu Z., Leong R. L., Chee M. W., Massar S. A. (2022). Bedtime procrastination and chronotype differentially predict adolescent sleep on school nights and non-school nights. Sleep Health.

[B48-behavsci-16-01327] Rique G. L., Fernandes Filho G. M., Ferreira A. D., de Sousa-Muñoz R. L. (2014). Relationship between chronotype and quality of sleep in medical students at the Federal University of Paraiba, Brazil. Sleep Science.

[B49-behavsci-16-01327] Shek T. L., Law M. Y. M. (2014). Parental behavioral control, parental psychological control and parent-child relational qualities: Relationships to Chinese adolescent risk behavior. Chinese adolescents in Hong Kong: Family life, psychological well-being and risk behavior.

[B50-behavsci-16-01327] Shi X., Fan F., Zeng Y., Zhu Y. (2019). Relationship between morningness–eveningness preference and depressive symptoms in adolescents: A chain mediating model. Chinese Journal of Clinical Psychology.

[B51-behavsci-16-01327] Shorey S., Ng E. D., Wong C. H. J. (2022). Global prevalence of depression and elevated depressive symptoms among adolescents: A systematic review and meta-analysis. The British Journal of Clinical Psychology.

[B52-behavsci-16-01327] Singh S., Lai C. H., Iderus N. H. M., Ghazali S. M., Ahmad L. C. R. Q., Cheng L. M., Nadzri M. N., Zulkifli A. A., Suppiah J., Zaki R. A. (2023). Prevalence and determinants of depressive symptoms among young adolescents in Malaysia: A cross-sectional study. Children.

[B53-behavsci-16-01327] Soenens B., Park S. Y., Mabbe E., Vansteenkiste M., Chen B., Van Petegem S., Brenning K. (2018). The moderating role of vertical collectivism in South-Korean adolescents’ perceptions of and responses to autonomy-supportive and controlling parenting. Frontiers in Psychology.

[B54-behavsci-16-01327] Soenens B., Vansteenkiste M. (2010). A theoretical upgrade of the concept of parental psychological control: Proposing new insights on the basis of self-determination theory. Developmental Review.

[B55-behavsci-16-01327] Soltani S., Noel M., Bernier E., Kopala-Sibley D. C. (2023). Pain and insomnia as risk factors for first lifetime onsets of anxiety, depression, and suicidality in adolescence. Pain.

[B56-behavsci-16-01327] Taillard J., Philip P., Chastang J. F., Bioulac B. (2004). Validation of Horne and Ostberg morningness-evening type questionnaire in a middle-aged population of French workers. Journal of Biological Rhythms.

[B57-behavsci-16-01327] Tang D., Wen Z. (2020). Statistical approaches for testing common method bias: Problems and suggestions. Journal of Psychological Science.

[B58-behavsci-16-01327] Tang T., Wang Y., Gong F., Shi K., Li X., Liu W., Chen N. (2024). The relationship between parenting styles and positive development of Chinese adolescents: A series of meta-analytic studies. Advances in Psychological Science.

[B59-behavsci-16-01327] Taylor B. J., Hasler B. P. (2018). Chronotype and mental health: Recent advances. Current Psychiatry Reports.

[B60-behavsci-16-01327] Thapar A., Eyre O., Patel V., Brent D. (2022). Depression in young people. Lancet.

[B61-behavsci-16-01327] Van den Berg J. F., Kivelä L., Antypa N. (2018). Chronotype and depressive symptoms in students: An investigation of possible mechanisms. Chronobiology International.

[B62-behavsci-16-01327] Wang F., Zhou Y., Hou X., Ni S., Xia X., Zhang T., Zhang Y., Li X., Wen K., Wang Y., Fu Y. (2025). The association of chronotype on depression in adolescents: The mediating role of sensation seeking and sleep quality. BMC Psychiatry.

[B63-behavsci-16-01327] Wang L., Wang K., Liu J. H., Wang Y. P. (2018). Altered default mode and sensorimotor network connectivity with striatal subregions in primary insomnia: A resting-state multi-band fMRI study. Frontiers in Neuroscience.

[B64-behavsci-16-01327] Wang M., Fan C., Zhou Z., Chen W. (2014). Parental conflict affects adolescents’ depression and social anxiety: Based on cognitive-contextual and emotional security theories. Acta Psychologica Sinica.

[B65-behavsci-16-01327] Wang Q., Pomerantz E. M., Chen H. (2007). The role of parents’ control in early adolescents’ psychological functioning: A longitudinal investigation in the United States and China. Child Development.

[B66-behavsci-16-01327] Wen Z., Huang B., Tang D. (2018). Questionnaire data modeling before analysis. Psychological Science.

[B67-behavsci-16-01327] Wittmann M., Dinich J., Merrow M., Roenneberg T. (2006). Social jetlag: Misalignment of biological and social time. Chronobiology International.

[B68-behavsci-16-01327] Xu X., Deng C., Liu M. (2020). Parents’ academic involvement and negative emotions in high school students: The mediating role of parent child relationship and the moderating role of parental psychological control. Journal of Psychological Science.

[B69-behavsci-16-01327] Ye S., Yang Y., Lu X., Jin H., Li J., Liu H., Liu L. (2025). Association of sleep and circadian rhythm disruption with co-occurring depressive and anxiety symptoms among primary and secondary school students. Chinese Journal of School Health.

[B70-behavsci-16-01327] Yılmaz H., Büyükcebeci A. (2019). Bazı pozitif psikoloji kavramları açısından helikopter ebeveyn tutumlarının sonuçları. Turkish Psychological Counseling and Guidance Journal.

[B71-behavsci-16-01327] Yue Y., Hong L., Guo L., Gao X., Deng J., Huang J., Huang G., Lu C. (2015). Gender differences in the association between cigarette smoking, alcohol consumption and depressive symptoms: A cross-sectional study among Chinese adolescents. Scientific Reports.

[B72-behavsci-16-01327] Zhang Y., Jin Z., Li S., Xu H., Wan Y., Tao F. (2023). Relationship between chronotype and mental behavioral health among adolescents: A cross-sectional study based on the social ecological system. BMC Psychiatry.

[B73-behavsci-16-01327] Zhong C. Y. (2023). A review of parenting styles. Advances in Social Sciences.

[B74-behavsci-16-01327] Zhou H., Long L. (2004). Statistical remedies for common method biases. Advances in Psychological Science.

[B75-behavsci-16-01327] Zhou S. J., Zhang L. G., Wang L. L., Guo Z. C., Wang J. Q., Chen J. C., Liu M., Chen X., Chen J. X. (2020). Prevalence and socio-demographic correlates of psychological health problems in Chinese adolescents during the outbreak of COVID-19. European Child & Adolescent Psychiatry.

[B76-behavsci-16-01327] Zhu Y., Huang J., Yang M. (2022). Association between chronotype and sleep quality among Chinese college students: The role of bedtime procrastination and sleep hygiene awareness. International Journal of Environmental Research and Public Health.

[B77-behavsci-16-01327] Zou H., Wang X., Bao C., Sun H., Xia Q., Chen Z., Zhou H., Yan R., Hua L., Veniero D., Yao Z. (2025). Impact of chronotype on depression: The mediating roles of rumination and perceived stress. Chronobiology International.

[B78-behavsci-16-01327] Zubrick S. R., Hafekost J., Johnson S. E., Sawyer M. G., Patton G., Lawrence D. (2017). The continuity and duration of depression and its relationship to non-suicidal self-harm and suicidal ideation and behavior in adolescents 12–17. Journal of Affective Disorders.

