# Supplementary material for: The Relationship Between Chronotype and Depression Among Adolescents: The Mediating Role of Bedtime Procrastination and the Moderating Role of Parental Psychological Control
Source: Behav Sci (Basel). 2026 Aug 3;16(8):1327. doi: 10.3390/bs16081327 (PMC13510148; doi:10.3390/bs16081327)
Supplement: Supplementary file 1 [file behavsci-16-01327-s001.zip › behavsci-4332131-supplementary.pdf]

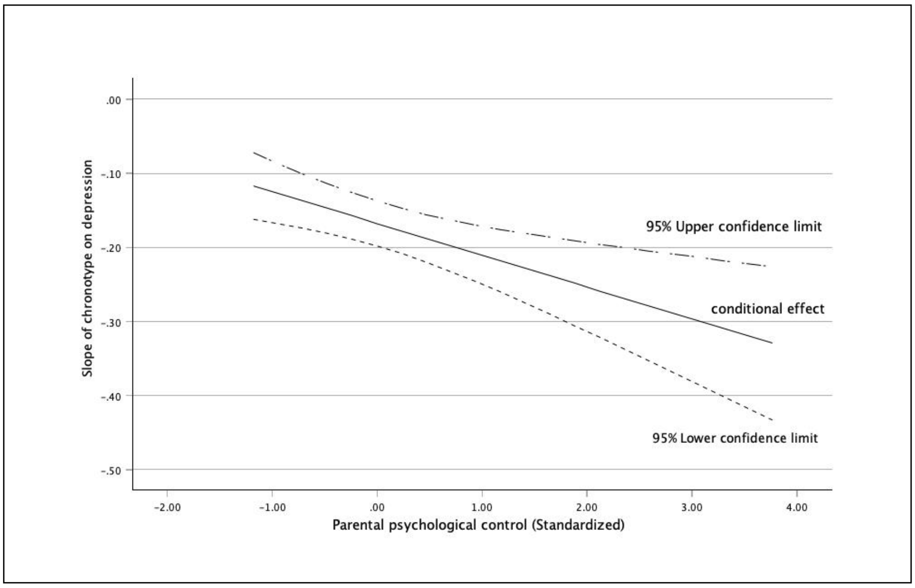

Figure S1. Johnson–Neyman plot of the conditional direct associat ion between chronotype and depression across levels of parental psychological control. The solid line represents the estimated conditional effect; the dashed lines represent the upper and lower bounds of the 95% bootstrap confidence interval. The association is statistically significant where the confidence interval does not contain zero.

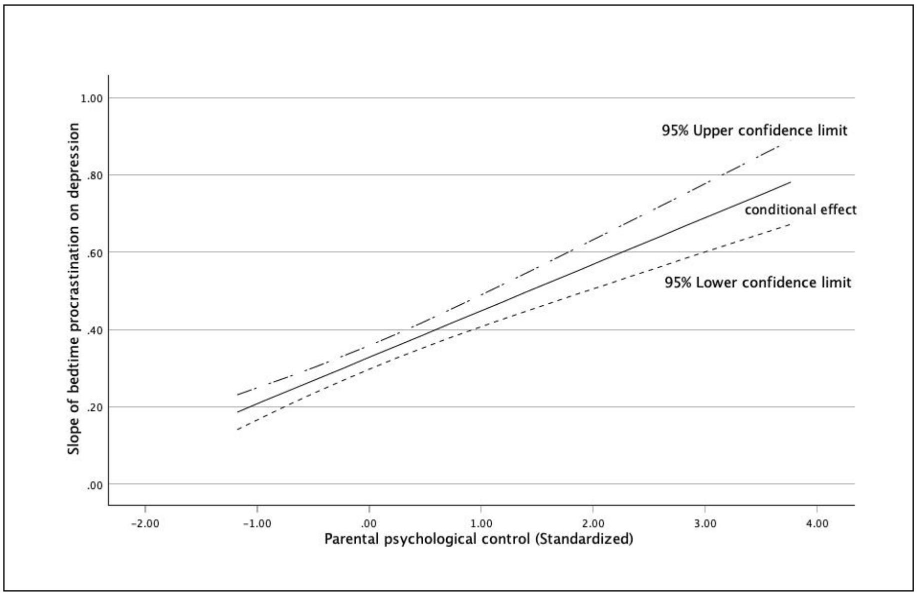

Figure S2. Johnson–Neyman plot of the conditional association between bedtime procrastination and depression across levels of parental psychological control. The solid line represents the estimated conditional effect; the dashed lines represent the upper and lower bounds of the 95% bootstrap confidence interval. The association is statistically significant where the confidence interval does not contain zero.

Table S1. Conditional indirect effects at different percentiles of parental psychological control.

Percentile of PPC	Effect	95% CI	
		Boot LLCI	Boot ULCI
25th percentile	−0.141	−0.164	−0.118
50th percentile	−0.187	−0.209	−0.169
75th percentile	−0.235	−0.263	−0.208

Notes: PPC, parental psychological control; Boot LLCI and Boot ULCI refer to the lower and upper limits of the 95% confidence interval of the indirect effects estimated by the percentile bootstrap method with deviation correction respectively. Bootstrap sample size=5000.
